# Calcium channel blockers do not protect against saturated fatty acid-induced ER stress and apoptosis in human pancreatic β-cells

**DOI:** 10.1186/s12986-021-00597-6

**Published:** 2021-07-17

**Authors:** Jan Šrámek, Vlasta Němcová, Jan Kovář

**Affiliations:** Department of Biochemistry, Cell and Molecular Biology and Center for Research of Diabetes, Metabolism, and Nutrition, Third Faculty of Medicine, Charles University, Prague, Czech Republic

**Keywords:** Apoptosis, Calcium influx, Diazoxide, Fatty acids, NES2Y, Nifedipine, Pancreatic β-cells, Type 2 diabetes mellitus, Verapamil, 1.1B4

## Abstract

It was evidenced that saturated fatty acids (FAs) have a detrimental effect on pancreatic β-cells function and survival, leading to endoplasmic reticulum (ER) calcium release, ER stress, and apoptosis. In the present study, we have tested the effect of three calcium influx inhibitors, i.e., diazoxide, nifedipine, and verapamil, on the apoptosis-inducing effect of saturated stearic acid (SA) in the human pancreatic β-cell lines NES2Y and 1.1B4. We have demonstrated that the application of all three calcium influx inhibitors tested has no inhibitory effect on SA-induced ER stress and apoptosis in both tested cell lines. Moreover, these inhibitors have pro-apoptotic potential per se at higher concentrations. Interestingly, these findings are in contradiction with those obtained with rodent cell lines and islets. Thus our data obtained with human β-cell lines suggest that the prospective usage of calcium channel blockers for prevention and therapy of type 2 diabetes mellitus, developed with the contribution of the saturated FA-induced apoptosis of β-cells, seems rather unlikely.

**Supplementary Information:**

The online version contains supplementary material available at 10.1186/s12986-021-00597-6.

## Background

Increased levels of saturated fatty acids (FAs) in the blood are considered to be one of the main factors responsible for pancreatic β-cell death in type 2 diabetes (T2DM) [1–5]. Our previous studies, as well as other studies, have shown that saturated FAs (e.g., stearic and palmitic acid) induce endoplasmic reticulum (ER) stress and apoptosis in pancreatic β-cells [3–9].

Increases in cytosolic calcium in β-cells directly stimulate insulin vesicle exocytosis, as well as initiate multiple signalling pathways which regulate a number of important cellular processes within the β-cell [10]. Saturated FAs were reported to stimulate calcium influx and mobilize calcium from ER pools in pancreatic β-cells [11–13]. The exact mechanisms are not clear; however, activation of G protein-coupled receptor 40 (GPR40) by FAs leading to inositol-1,4,5-trisphosphate (IP3) production may be involved here. The release of calcium from the ER occurs primarily via IP3-regulated IP3 receptors (IP3R) but may be also mediated by ryanodine receptors (RyRs). Rodent as well as human β-cells express all IP3R isoforms and importantly, IP3Rs are upregulated in human islets from patients with T2DM, leading to mitochondrial dysfunction and pancreatic β-cell failure [14]. Mitochondrial dysfunction may be promoted by FA-induced ER calcium release [15]. All RyRs family members, which represent LTCC (L-type voltage-gated calcium channels), seem to be also expressed in human islets [16]. Since β-cells as secretory cells are very sensitive to ER homeostasis disturbances, calcium depletion can result in the accumulation of misfolded proteins in the ER and subsequently in an ER stress response [17] and apoptosis induction [18–21]. Taken together, these facts suggest that calcium influx regulation could be involved in FA-induced β-cell dysfunction and apoptosis.

It was documented in rodent β-cell lines and islets that some calcium chelators or blocking of calcium signalling have a protective effect on FA-induced β-cell apoptosis [12, 22–27]. Moreover, the protective effect of some calcium influx inhibitors against other pro-apoptotic treatments (e.g. streptozocin, high glucose) or the generally protective effect per se on β-cells was suggested in diabetic mice and patients [26, 28, 29]. Thus, regulation of calcium influx could represent a protective factor against impaired function and apoptosis induced by FAs in human pancreatic β-cells during the development of T2DM.

Diazoxide, nifedipine, and verapamil represent commonly used calcium channel blockers. Concerning the mechanisms of their effect, nifedipine and verapamil directly block LTCC while diazoxide binds to the K_ATP_ channel keeping it open which decreases membrane hyperpolarisation and activation of LTCC [30, 31]. These compounds are routinely used as a medication for several diseases such as hypertension, chronic angina, or ischemic disease [32]. It seems that calcium influx via LTCC mediates IP3Rs upregulation what may also increase intracellular calcium level [33, 34].

We hypothesize that calcium channel blockers may be protective against FA-induced apoptosis in human β-cell similarly as it was previously documented in rodent β-cells and islets [12, 22–27]. Therefore, we tested the effect of three calcium influx inhibitors, i.e. diazoxide, nifedipine, and verapamil, on the apoptosis-inducing effect of saturated stearic acid (SA) in the human pancreatic β-cell lines NES2Y and 1.1B4.

## Material and methods

### Materials

All chemicals were sourced from Sigma-Aldrich (St. Louis, MO, USA), unless otherwise stated. For the western blot analysis, the following primary and secondary antibodies were used: anti-cleaved caspase-3 (#9661), caspase-6 (#9761), anti-cleaved caspase-7 (#9491), anti-cleaved caspase-8 (#9496), anti-cleaved caspase-9 (#9505), anti-cleaved PARP (#5625), anti-BiP (#3177), anti-CHOP (#2895) from Cell Signaling Technology (Danvers, MA, USA); anti-caspase-2 (ab32021) from Abcam (Cambridge, UK); anti-actin (clone AC-40); HRP-linked goat anti-mouse (SA00001-1) and goat anti-rabbit (SA00001-2) secondary antibody from Proteintech Group (Rosemont, IL, USA). As calcium influx inhibitors, diazoxide (D9035), nifedipine (N7634), and verapamil (ab120140) from Abcam were used. Diazoxide and nifedipine were dissolved and diluted in DMSO while verapamil in distilled water. Concentrations in range 1–1000 µM for nifedipine and verapamil, and 3–3000 µM for diazoxide were used in order to detect the effect of the respective inhibitors. Selected concentrations are within the range used in similar studies [22–24, 26, 27]. All solutions were prepared and used according to manufacturers’ protocols.

### Cells and culture conditions

**Fig. 1:**
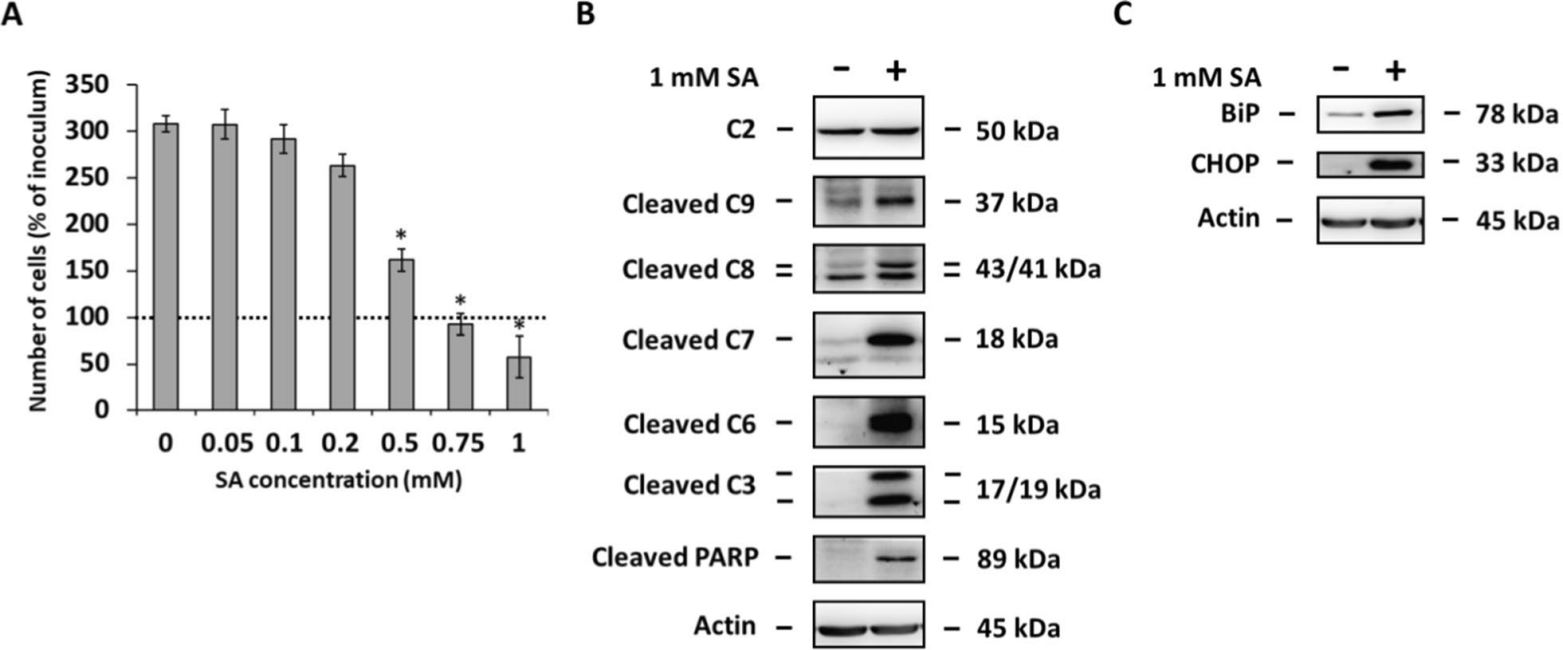
Dose-dependent effect of stearic acid (SA) on cell growth and viability in 1.1B4 cells (**A**). Effect of 1 mM SA on the level of caspase-2 (C2), cleaved caspase-9 (C9), cleaved caspase-8 (C8), cleaved caspase-7 (C7), cleaved caspase-6 (C6), cleaved caspase-3 (C3), and cleaved PARP in 1.1B4 cells, (**B**). Effect of 1 mM SA on the level of BiP and CHOP in 1.1B4 cells (**C**). Cells incubated without SA represented control cells. When assessing cell growth and viability (**A**), the number of living cells was determined after 48 h of incubation. Each column represents the mean of four separate cultures ± SEM. * p < 0.05 when comparing the number of control cells and cells treated with SA. The dotted line represents the number of cells of the inoculum. After 24 h of the incubation (**B, C**), the levels of individual proteins were determined using western blot analysis and the relevant antibodies (see “Materials and Methods”). Monoclonal antibody against human actin was used to confirm equal protein loading. The data shown were obtained in one representative experiment from at least three independent experiments

The human pancreatic β-cell lines NES2Y and 1.1B4 [35, 36] were used. NES2Y cells were derived from a patient with persistent hyperinsulinemic hypoglycemia of infancy [35] and were kindly provided by Dr. Roger F. James (University of Leicester, UK). 1.1B4 cells were generated by electrofusion of freshly isolated human pancreatic β-cells and the immortal human PANC-1 epithelial cell line [36] and were purchased from MERCK Millipore (# 10012801, Burlington, MA, USA). Cells were routinely maintained in an RPMI 1640-based culture medium [37]. A 1 mM concentration of SA was used to simulate an elevated level of SA in the blood [38, 39]. The stock solution containing SA bound to 10% BSA in a serum-free medium was prepared as described previously [3] and diluted to 1 mM concentration of SA and 2% BSA prior to experiments. SA/BSA molar ratios used in the experiments were lower than the ratios known to exceed BSA's binding capacity [40]. A 1 mM concentration of SA was established as apoptosis-inducing in both cell lines [3] (Fig. 1a). In experiments, a defined serum-free medium was used [41]. Our previous studies showed that SA, at a concentration of 1 mM, induces endoplasmic reticulum stress and apoptosis in most NES2Y cells within 24 h of application [7, 8, 42–44]. Therefore, all assessments were performed within 24 h after SA application except for the assessment of cell growth and viability. Prior, during, and at the end of each experiment, cell condition was visually checked.

### Assessment of cell growth and viability

Cells were seeded at a concentration of 2 × 10^4^ cells/100 μl of culture media into the wells of the 96-well plate. After a 24-h pre-incubation period (allowing cells to attach), the culture medium was replaced with a serum-free medium containing 2% BSA with or without SA and/or the respective calcium influx inhibitor (various concentrations were used) and/or 0.1% DMSO as the vehicle. After 48 h of incubation, cell images were obtained and the number of living cells was determined using a hemocytometer counting, after staining with trypan blue.

### Western blot analysis

Cells (approximately 250,000 cells per sample) were seeded into the wells of the 12-well plate. After a 24-h pre-incubation period (allowing cells to attach), the culture medium was replaced with a serum-free medium containing 2% BSA with or without SA and with or without the respective calcium influx inhibitor (various concentrations were used). The control medium contained 2% BSA only or 2% BSA with 0.1% DMSO or distilled water as a vehicle. After 24-h of incubation, cells were washed twice with PBS and lysed by 50 μl RIPA lysis buffer (containing 0.5 μl protease inhibitor cocktail) and harvested. Cell lysates were then centrifuged (18,000 g, 20 min, 4 °C). Supernatants were collected and frozen at -80 °C until further analysis. Total protein content was determined by the bicinchoninic acid assay [45]. Samples (6.5 μl) containing 15 μg of proteins were mixed with 6.5 μl of sample loading buffer (0.125 mM Tris–HCl, pH 6.8, 10% glycerol, 4% SDS, 250 mM DTT, 0.004% bromphenol blue), heated for 10 min at 95 °C. SDS-PAGE was performed as described previously [46, 47] with minor modifications. Briefly, proteins were separated on 12% polyacrylamide gel (4% polyacrylamide stacking gel) at 30 mA and then blotted onto 0.2 μm nitrocellulose transfer membrane (Protran BA83, Schleicher-Schuell, Dassel, Germany) for 2 h at 0.25 A using a Mini-Protean 3 apparatus (Bio-Rad, Hercules, CA). The membrane was blocked with 5% BSA in TBS (100 mM Tris–HCl, 150 mM NaCl, pH 7.5) for 20 min, washed with 0.1% Tween-20/TBS three times, and then incubated with the primary antibody in 0.1% Tween-20/TBS containing 1% BSA overnight at 4 °C. All primary antibodies were used in a 1:1,000 dilution. The chemiluminescent signal was detected using a Carestream Gel Logic 4000 PRO Imaging System equipped with Carestream Molecular Imaging Software (Carestream Health, New Haven, CT, USA), which was used for image acquisition.

### Data analysis

The statistical significance of observed differences was determined using the Student’s t-test. p < 0.05 was considered statistically significant.

## Results

### Effect of stearic acid on growth and viability, expression of ER stress markers and apoptosis induction

Our previous, as well as current data, show that SA (1 mM) induces ER stress and apoptosis in NES2Y [3, 7, 8, 42, 43] as well as in 1.1B4 (Fig. 1) cells. The expression of the main ER stress markers, i.e., proteins immunoglobulin heavy chain-binding protein (BiP) and CCAAT enhancer-binding protein homologous protein (CHOP), was increased due to SA treatment in both cell lines [8] (Fig. 1c). SA-induced also activation (cleavage) of the main markers of ongoing apoptosis, i.e., caspases-3, -6, -7, -8, -9 and protein poly ADP-ribose polymerase (PARP) (substrate of executioner caspases) in both cell lines [7, 8, 42, 43] (Fig. 1a, b). In NES2Y cells, caspase-2 was also activated by SA, but it seems not to play a key role in SA-induced apoptosis [7]. In 1.1B4 cells, caspase-2 was not activated due to SA treatment (Fig. 1B).

### Effect of diazoxide on stearic acid-induced apoptosis and expression of ER stress markers BiP and CHOP

**Fig. 2:**
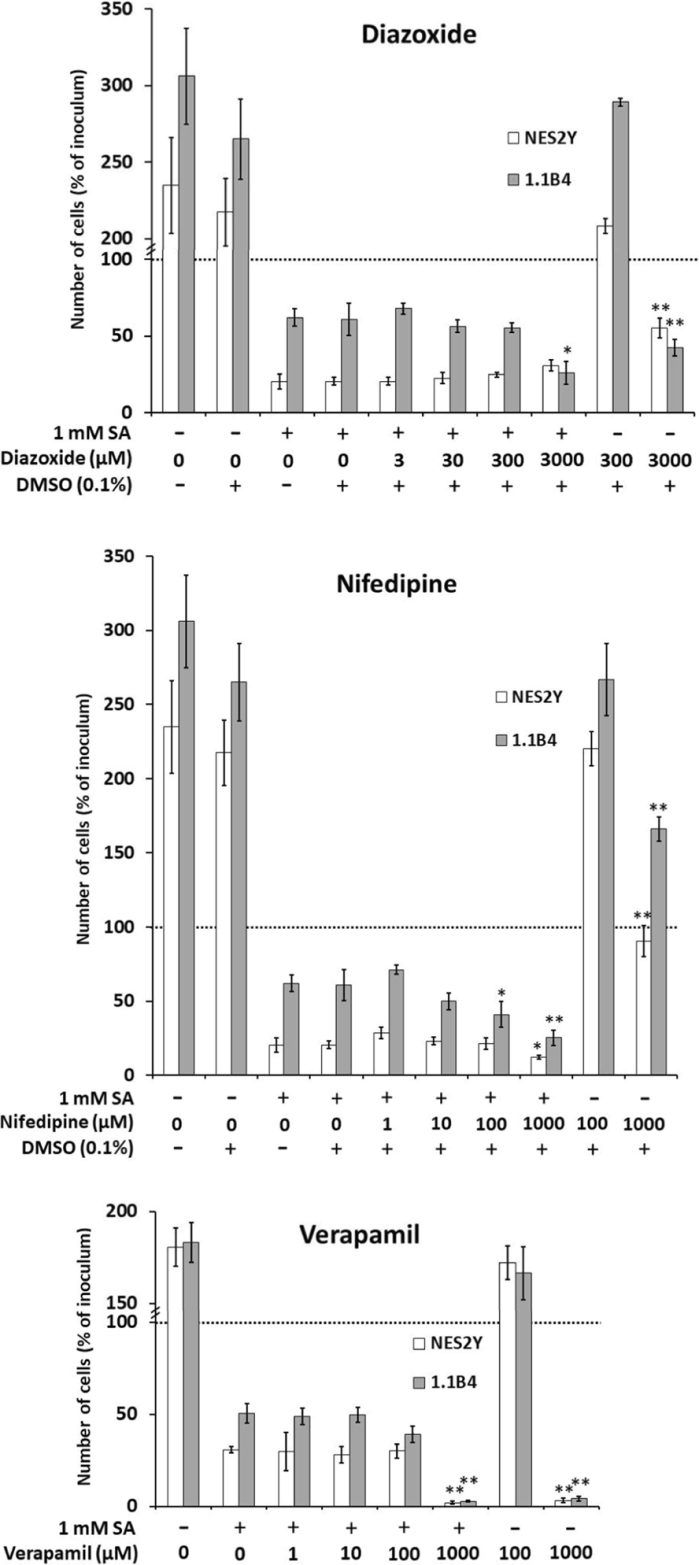
Dose-dependent effect of diazoxide, nifedipine, and verapamil on the effect of stearic acid (SA) on cell growth and viability in NES2Y and 1.1B4 cells. The data shown were obtained in one representative experiment of at least three independent experiments. The number of living cells was determined after 48 h of incubation. Each column represents the mean of at least four separate cultures ± SEM. * p < 0.05, ** p < 0.001 when comparing the number of cells treated with the respective inhibitor together with SA and cells with SA only. The dotted line represents the number of cells of the inoculum. DMSO was used as vehicle

Diazoxide at a concentration of 3, 30, 300, and 3,000 µM had no significant effect on the effect of SA on β-cell growth and viability compared to cells treated with SA only in NES2Y as well as 1.1B4 cells except for 3,000 µM concentration in 1.1B4 cells which significantly potentiated the effect of SA. Diazoxide per se had no significant effect on β-cells growth and viability at a concentration of 300 µM. However, 3,000 µM concentration significantly decreased β-cell growth and viability in both cell lines (Fig. 2). The cell phenotype correlated with the found effect of the respective treatment on β-cell viability (Additional file 1: Figure S1).

**Fig. 3:**
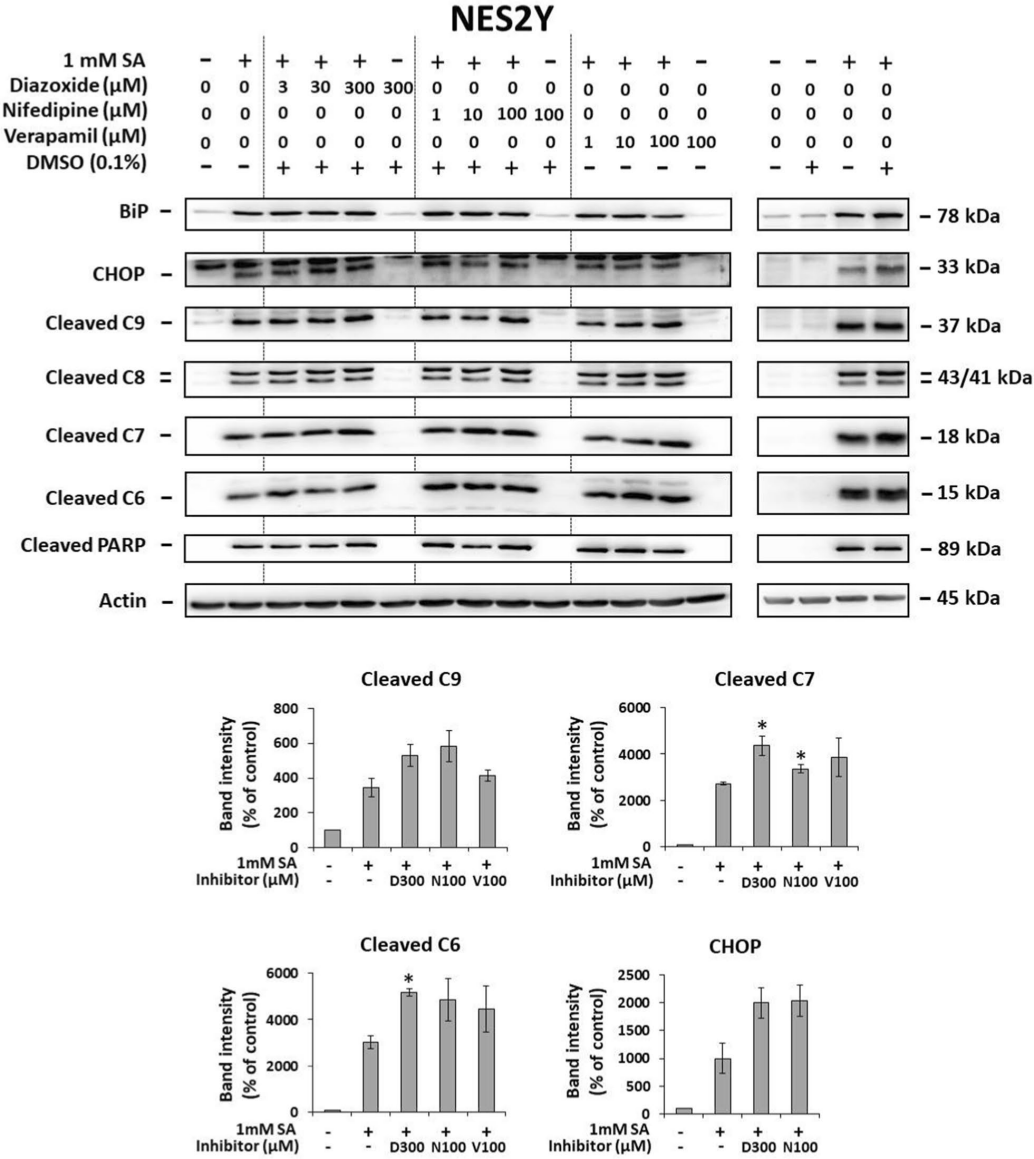

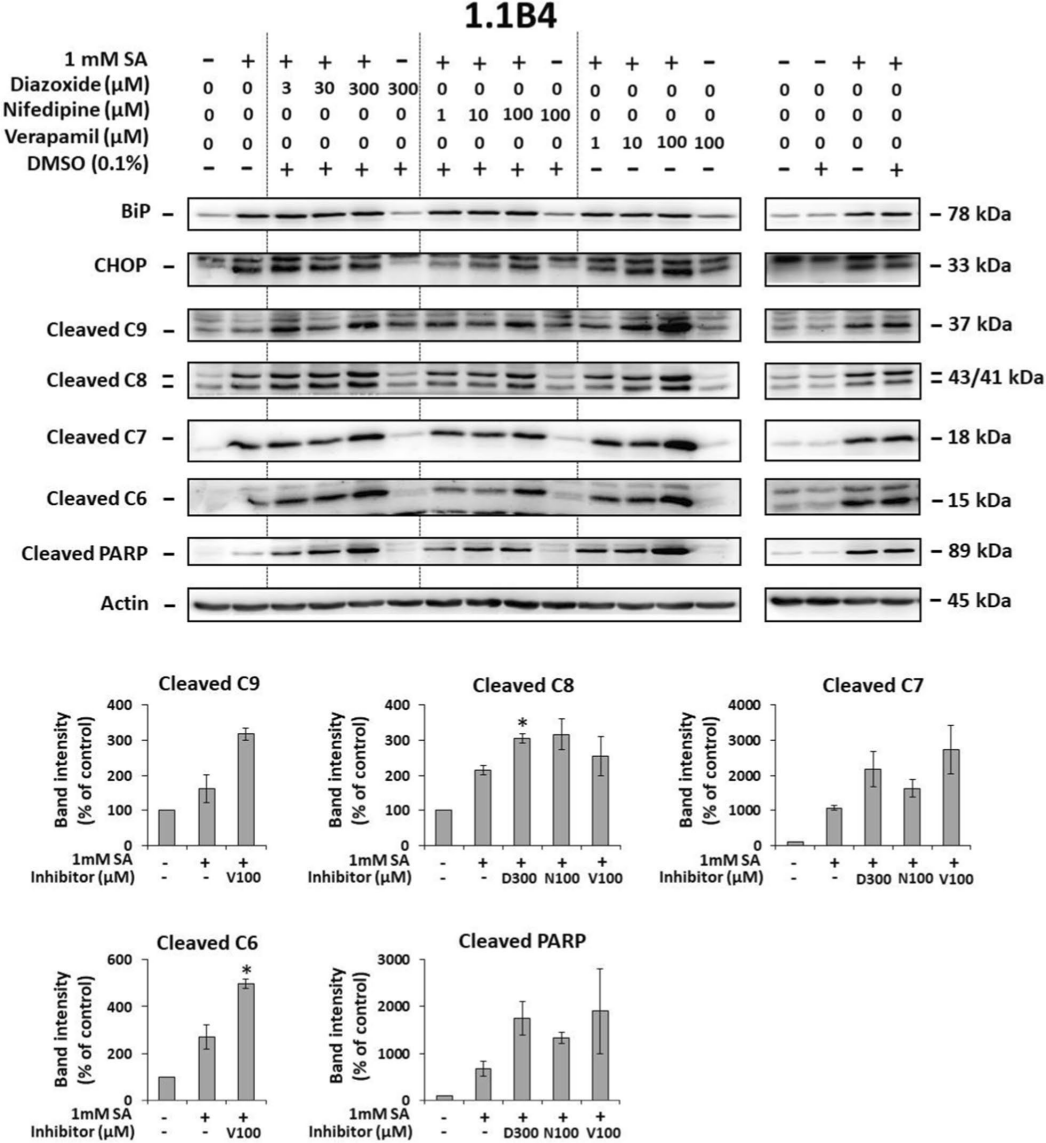
Effect of diazoxide, nifedipine, and verapamil on the effect of stearic acid (SA) on the level of cleaved caspase-9 (C9), caspase-8 (C8), caspase-7 (C7), caspase-6 (C6), and cleaved PARP (markers of apoptosis) and on the level of BiP and CHOP (markers of ER stress) in NES2Y and 1.1B4 cells. After 24 h of incubation, the levels of individual proteins were determined using western blot analysis and the relevant antibodies (see “Materials and Methods”). Monoclonal antibody against human actin was used to confirm equal protein loading. The data shown were obtained in one representative experiment from two till six independent experiments. DMSO was used as vehicle. Densitometric analysis of key data from Western blotting where perceptible change in the expression was detected is also shown. Each column represents the mean of at least two experimental values ± SEM. * p < 0.05 when comparing the effect of respective inhibitor + 1 mM SA with the effect of 1 mM SA applied alone. D300 means 300 µM diazoxide, N100 means 100 µM nifedipine and V100 means 100 µM verapamil

Diazoxide at a concentration of 3 and 30 µM had no effect on SA-induced caspases activation by their cleavage and PARP cleavage as well as on SA-induced expression of BiP in NES2Y cells. However, 300 µM diazoxide increased the SA-induced cleavage of caspase-6, -7 and -9, and expression of CHOP. Diazoxide at the highest concentration used had no effect on the tested molecules per se (Fig. 3).

In 1.1B4 cells, diazoxide increased SA-induced cleavage of caspase, -7, -8, and PARP, and had no significant effect on SA-induced expression of the other tested molecules. Diazoxide per se only slightly increased cleavage of caspase-7 among tested molecules (Fig. 3).

### Effect of nifedipine on stearic acid-induced apoptosis and expression of ER stress markers BiP and CHOP

Nifedipine at a concentration of 1, 10, and 100 µM had no significant effect on the effect of SA on β-cell growth and viability compared to cells treated with SA only in NES2Y as well as 1.1B4 cells except for 100 µM concentration in 1.1B4 cells, which significantly potentiated the effect of SA. Nifedipine at a concentration of 1,000 µM then significantly potentiated the effect of SA in both cell lines. Nifedipine per se had no significant effect on β-cells growth and viability at a concentration of 100 µM. However, 1,000 µM concentration significantly decreased β-cells growth and viability in both cell lines (Fig. 2). The cell phenotype correlated with the found effect of the respective treatment on β-cell viability (Additional file 1: Figure S1).

In NES2Y cells, nifedipine at a concentration of 1 and 10 µM had no significant effect on SA-induced cleavage of caspases and PARP as well as on SA-induced expression of BiP. It only increased cleavage of caspase-6, -7 and -9, and expression of CHOP when 100 µM nifedipine was used. Nifedipine had no effect per se (Fig. 3).

Similar results were obtained in 1.1B4 cells. Nifedipine at a concentration of 1 and 10 µM had no significant effect on SA-induced cleavage of caspases and PARP as well as on SA-induced expression of BiP and CHOP while 100 µM concentration increased SA-induced cleavage of caspase-7, -8 and PARP. Nifedipine per se slightly increased cleavage of caspase-6 and -7 (Fig. 3).

### Effect of verapamil on stearic acid-induced apoptosis and expression of ER stress markers BiP and CHOP

Verapamil at a concentration of 1, 10, and 100 µM had no significant effect on the effect of SA on β-cell growth and viability compared to cells treated with SA in NES2Y as well as 1.1B4 cells except for 100 µM concentration in 1.1B4 cells, which significantly potentiated the effect of SA. Verapamil at a concentration of 1,000 µM then significantly potentiated the effect of SA in both cell lines. Verapamil per se had no significant effect on β-cells growth and viability at a concentration of 100 µM concentration; however, 1,000 µM concentration significantly decreased β-cells growth and viability in both cell lines (Fig. 2). The phenotype of the cells corresponded with the found effect of the respective treatment on β-cell viability (Additional file 1: Figure S1).

Verapamil at a concentration of 1and 10 µM had no significant effect on SA-induced cleavage of caspase-8, -9, and PARP as well as on SA-induced expression of BiP and CHOP in NES2Y cells. It only slightly increased SA-induced caspase-6, -7 and -9 cleavage. Verapamil per se had no effect (Fig. 3).

In 1.1B4 cells, verapamil increased SA-induced cleavage of caspases and PARP while CHOP and BiP expression was not changed. Verapamil per se increased among tested molecules expression of BiP and CHOP, and slightly also cleavage of caspase-6 and -7 (Fig. 3).

## Discussion

As well as other authors, we have shown that saturated FAs (e.g., stearic and palmitic acid) induce ER stress and apoptosis in pancreatic β-cells [1–5]. It was suggested that calcium influx regulation is involved in FA-induced β-cell dysfunction and apoptosis [e.g. [11, 13, 17, 40]]. It was shown in rodent cell lines and islets that some calcium chelators or calcium signal blockers have a protective effect on FA-induced β-cell apoptosis [22–24, 26, 27]. Thus, regulation of calcium influx could represent a protective factor against FA-induced dysfunction and apoptosis in human pancreatic β-cells during the development of type 2 diabetes mellitus. The aim of this study was to test the effect of three calcium influx inhibitors, i.e., diazoxide, nifedipine, and verapamil, on the apoptosis-inducing effect of saturated stearic acid (SA) in the human pancreatic β-cell lines NES2Y and 1.1B4.

Our data showed that the application of these three calcium influx inhibitors had no inhibitory effect on SA-induced ER stress and apoptosis in both human cell lines tested. Moreover, at higher concentrations, they were pro-apoptotic per se (see Figs. 2, 3 and Additional file 1: Fig. S1). It suggests that in human pancreatic β-cells, calcium influx stimulation is not involved in saturated FA-induced apoptosis. Interestingly, our findings are in contradiction with those obtained with rodent models where authors documented the protective effect of the same compounds against saturated FA-induced ER stress and apoptosis [22–24, 26, 27]. They used similar or the same concentrations of the respective calcium channel blockers.

In these studies, palmitate was used as saturated FA. Thus one can speculate that obtained different effects of calcium influx inhibitors on saturated FA-induced ER stress and apoptosis may be due to differences in mechanisms by which palmitate and stearate induce these processes in β-cells, e.g., the different effect of calcium signalling or metabolism on these processes. However, mechanisms mediating pro-apoptotic effects of saturated FAs were not systematically studied for individual types of saturated FAs. Thus possible differences in these mechanisms are not known. Nevertheless, we have data showing no effect of all the three calcium influx inhibitors on palmitate-induced ER stress and apoptosis in 1.1B4 human cells as well (our unpublished data).

In some of the mentioned studies, where tested compounds were protective against saturated FA-induced ER stress and apoptosis, a lower concentration (usually 0.5 mM) of palmitate was used. It may indicate that used channel blockers are not effective against higher concentrations of saturated FAs. However, we tested the effect of these compounds also against ER stress and apoptosis induced by 0.5 and 0.75 mM SA. We obtained the same negative results as in the case of 1 mM (our unpublished data).

To conclude, it seems that calcium channel blockers tested are unable to attenuate saturated FA-induced ER stress and apoptosis in human pancreatic β-cells. According to these findings, their possible benefit in the prevention and therapy of type 2 diabetes mellitus development with a contribution of saturated FA-induced apoptosis of β-cells is rather unlikely. However, we did not test the effect of calcium influx inhibitors on insulin secretion. Thus there is still a possibility that these compounds may exert a certain protective effect in human β-cells via inhibition of saturated FA-induced impairment of insulin secretion as found in rodent β-cells [27, 48]. Tested compounds themselves (especially verapamil) had when using higher concentrations even pro-apoptotic effect on β-cells. This finding may have a certain impact on calcium channel blockers' usage in the therapy of other diseases, e.g. ischemic disease, chronic angina, or hypertension. While the concentrations of diazoxide and verapamil which had the pro-apoptotic effect on pancreatic β-cells (Fig. 2) are 20 times and more higher than the concentrations measured in serum of patients during e.g. hypertension treatment [49, 50], the pro-apoptotic concentration of nifedipine (100 µM) is similar to concentration present in the serum of these patients [51]. However, more studies using human pancreatic β-cells are needed to verify our results.

## Supplementary Information

**Additional file 1: Figure S1**. In vitro images of NES2Y and 1.1B4 β-cells after the treatment.

## Data Availability

The datasets used and/or analyzed during the current study are available from the corresponding author on reasonable request.

## Abbreviations

BiP: Immunoglobulin heavy chain-binding protein
BSA: Bovine serum albumin
CHOP: CCAAT enhancer-binding protein homologous protein
ER: Endoplasmic reticulum
FAs: Fatty acids
GPR40: G protein-coupled receptor
IP3R: IP3-regulated IP3 receptors
IP3: Inositol-1,4,5-triphosphate
LTCC: L-type calcium channel
PARP: Protein poly ADP-ribose polymerase
RyRs: Ryanodine receptors
SA: Stearic acid
T2DM: Type 2 diabetes mellitus

## References

[1] Lupi R, Dotta F, Marselli L, Del Guerra S, Masini M, Santangelo C, Patane G, Boggi U, Piro S, Anello M (2002). Prolonged exposure to free fatty acids has cytostatic and pro-apoptotic effects on human pancreatic islets: evidence that beta-cell death is caspase mediated, partially dependent on ceramide pathway, and Bcl-2 regulated. Diabetes.

[2] Azevedo-Martins AK, Monteiro AP, Lima CL, Lenzen S, Curi R (2006). Fatty acid-induced toxicity and neutral lipid accumulation in insulin-producing RINm5F cells. Toxicol In Vitro.

[3] Furstova V, Kopska T, James RF, Kovar J (2008). Comparison of the effect of individual saturated and unsaturated fatty acids on cell growth and death induction in the human pancreatic beta-cell line NES2Y. Life Sci.

[4] Maedler K, Oberholzer J, Bucher P, Spinas GA, Donath MY (2003). Monounsaturated fatty acids prevent the deleterious effects of palmitate and high glucose on human pancreatic beta-cell turnover and function. Diabetes.

[5] Welters HJ, Diakogiannaki E, Mordue JM, Tadayyon M, Smith SA, Morgan NG (2006). Differential protective effects of palmitoleic acid and cAMP on caspase activation and cell viability in pancreatic beta-cells exposed to palmitate. Apoptosis.

[6] Diakogiannaki E, Welters HJ, Morgan NG (2008). Differential regulation of the endoplasmic reticulum stress response in pancreatic beta-cells exposed to long-chain saturated and monounsaturated fatty acids. J Endocrinol.

[7] Nemcova-Furstova V, Balusikova K, Sramek J, James RF, Kovar J (2013). Caspase-2 and JNK activated by saturated fatty acids are not involved in apoptosis induction but modulate ER stress in human pancreatic beta-cells. Cell Physiol Biochem.

[8] Sramek J, Nemcova-Furstova V, Pavlikova N, Kovar J. Effect of saturated stearic acid on MAP kinase and ER stress signaling pathways during apoptosis induction in human pancreatic beta-cells is inhibited by unsaturated oleic acid. Int J Mol Sci. 2017;18:2313. 10.3390/ijms18112313 PMC5713282 29099080

[9] Welters HJ, Tadayyon M, Scarpello JHB, Smith SA, Morgan NG (2004). Mono-unsaturated fatty acids protect against β-cell apoptosis induced by saturated fatty acids, serum withdrawal or cytokine exposure. FEBS Lett.

[10] Sabatini PV, Speckmann T, Lynn FC (2019). Friend and foe: beta-cell Ca(2+) signaling and the development of diabetes. Mol Metab.

[11] Gwiazda KS, Yang TL, Lin Y, Johnson JD (2009). Effects of palmitate on ER and cytosolic Ca2+ homeostasis in beta-cells. Am J Physiol Endocrinol Metab.

[12] Ly LD, Ly DD, Nguyen NT, Kim JH, Yoo H, Chung J, Lee MS, Cha SK, Park KS (2020). Mitochondrial Ca(2+) uptake relieves palmitate-induced cytosolic Ca(2+) overload in MIN6 cells. Mol Cells.

[13] Remizov O, Jakubov R, Dufer M, Krippeit Drews P, Drews G, Waring M, Brabant G, Wienbergen A, Rustenbeck I, Schofl C (2003). Palmitate-induced Ca2+-signaling in pancreatic beta-cells. Mol Cell Endocrinol.

[14] Shu J, Gambardella J, Sorriento D, Santulli G (2018). Mechanistic role of IP3R calcium release channel in pancreatic beta-cell function. Diabetes.

[15] Egnatchik RA, Leamy AK, Jacobson DA, Shiota M, Young JD (2014). ER calcium release promotes mitochondrial dysfunction and hepatic cell lipotoxicity in response to palmitate overload. Mol Metab.

[16] Dror V, Kalynyak TB, Bychkivska Y, Frey MH, Tee M, Jeffrey KD, Nguyen V, Luciani DS, Johnson JD (2008). Glucose and endoplasmic reticulum calcium channels regulate HIF-1beta via presenilin in pancreatic beta-cells. J Biol Chem.

[17] Back SH, Kaufman RJ (2012). Endoplasmic reticulum stress and type 2 diabetes. Annu Rev Biochem.

[18] Cnop M, Ladriere L, Igoillo-Esteve M, Moura RF, Cunha DA (2010). Causes and cures for endoplasmic reticulum stress in lipotoxic beta-cell dysfunction. Diabetes Obes Metab.

[19] Cunha DA, Hekerman P, Ladriere L, Bazarra-Castro A, Ortis F, Wakeham MC, Moore F, Rasschaert J, Cardozo AK, Bellomo E (2008). Initiation and execution of lipotoxic ER stress in pancreatic beta-cells. J Cell Sci.

[20] Karaskov E, Scott C, Zhang L, Teodoro T, Ravazzola M, Volchuk A (2006). Chronic palmitate but not oleate exposure induces endoplasmic reticulum stress, which may contribute to INS-1 pancreatic beta-cell apoptosis. Endocrinology.

[21] Tabas I, Ron D (2011). Integrating the mechanisms of apoptosis induced by endoplasmic reticulum stress. Nat Cell Biol.

[22] Choi SE, Kim HE, Shin HC, Jang HJ, Lee KW, Kim Y, Kang SS, Chun J, Kang Y (2007). Involvement of Ca2+-mediated apoptotic signals in palmitate-induced MIN6N8a beta cell death. Mol Cell Endocrinol.

[23] Rorsman P, Arkhammar P, Bokvist K, Hellerström C, Nilsson T, Welsh M, Welsh N, Berggren PO (1989). Failure of glucose to elicit a normal secretory response in fetal pancreatic beta cells results from glucose insensitivity of the ATP-regulated K+ channels. Proc Natl Acad Sci USA.

[24] Sargsyan E, Ortsater H, Thorn K, Bergsten P (2008). Diazoxide-induced beta-cell rest reduces endoplasmic reticulum stress in lipotoxic beta-cells. J Endocrinol.

[25] Vogel J, Yin J, Su L, Wang SX, Zessis R, Fowler S, Chiu CH, Wilson AC, Chen A, Zecri F (2020). A phenotypic screen identifies calcium overload as a key mechanism of beta-cell glucolipotoxicity. Diabetes.

[26] Xu G, Chen J, Jing G, Shalev A (2012). Preventing β-cell loss and diabetes with calcium channel blockers. Diabetes.

[27] Zhou Y, Sun P, Wang T, Chen K, Zhu W, Wang H (2015). Inhibition of calcium influx reduces dysfunction and apoptosis in lipotoxic pancreatic beta-cells via regulation of endoplasmic reticulum stress. PLoS ONE.

[28] Huang Q, Bu S, Yu Y, Guo Z, Ghatnekar G, Bu M, Yang L, Lu B, Feng Z, Liu S, Wang F (2007). Diazoxide prevents diabetes through inhibiting pancreatic beta-cells from apoptosis via Bcl-2/Bax rate and p38-beta mitogen-activated protein kinase. Endocrinology.

[29] Guldstrand M, Grill V, Björklund A, Lins PE, Adamson U (2002). Improved beta cell function after short-term treatment with diazoxide in obese subjects with type 2 diabetes. Diabetes Metab.

[30] Doyle ME, Egan JM (2003). Pharmacological agents that directly modulate insulin secretion. Pharmacol Rev.

[31] Striessnig J, Ortner NJ, Pinggera A (2015). Pharmacology of L-type calcium channels: novel drugs for old targets?. Curr Mol Pharmacol.

[32] Elmslie KS (2004). Calcium channel blockers in the treatment of disease. J Neurosci Res.

[33] Genazzani AA, Carafoli E, Guerini D (1999). Calcineurin controls inositol 1,4,5-trisphosphate type 1 receptor expression in neurons. Proc Natl Acad Sci USA.

[34] Abou-Saleh H, Pathan AR, Daalis A, Hubrack S, Abou-Jassoum H, Al-Naeimi H, Rusch NJ, Machaca K (2013). Inositol 1,4,5-trisphosphate (IP3) receptor up-regulation in hypertension is associated with sensitization of Ca2+ release and vascular smooth muscle contractility. J Biol Chem.

[35] Macfarlane WM, Cragg H, Docherty HM, Read ML, James RF, Aynsley-Green A, Docherty K (1997). Impaired expression of transcription factor IUF1 in a pancreatic beta-cell line derived from a patient with persistent hyperinsulinaemic hypoglycaemia of infancy (nesidioblastosis). FEBS Lett.

[36] McCluskey JT, Hamid M, Guo-Parke H, McClenaghan NH, Gomis R, Flatt PR (2011). Development and functional characterization of insulin-releasing human pancreatic beta cell lines produced by electrofusion. J Biol Chem.

[37] Musílková J, Kovár J (2001). Additive stimulatory effect of extracellular calcium and potassium on non-transferrin ferric iron uptake by HeLa and K562 cells. Biochim Biophys Acta.

[38] Abdelmagid SA, Clarke SE, Nielsen DE, Badawi A, El-Sohemy A, Mutch DM, Ma DW (2015). Comprehensive profiling of plasma fatty acid concentrations in young healthy Canadian adults. PLoS ONE.

[39] Lagerstedt SA, Hinrichs DR, Batt SM, Magera MJ, Rinaldo P, McConnell JP (2001). Quantitative determination of plasma c8–c26 total fatty acids for the biochemical diagnosis of nutritional and metabolic disorders. Mol Genet Metab.

[40] Cnop M, Hannaert JC, Hoorens A, Eizirik DL, Pipeleers DG (2001). Inverse relationship between cytotoxicity of free fatty acids in pancreatic islet cells and cellular triglyceride accumulation. Diabetes.

[41] Kovar J, Franek F (1989). Growth-stimulating effect of transferrin on a hybridoma cell line: relation to transferrin iron-transporting function. Exp Cell Res.

[42] Nemcova-Furstova V, James RFL, Kovar J (2011). Inhibitory effect of unsaturated fatty acids on saturated fatty acid-induced apoptosis in human pancreatic beta-cells: activation of caspases and ER stress induction. Cell Physiol Biochem.

[43] Sramek J, Nemcova-Furstova V, Balusikova K, Daniel P, Jelinek M, James RF, Kovar J (2016). p38 MAPK is activated but does not play a key role during apoptosis induction by saturated fatty acid in human pancreatic beta-cells. Int J Mol Sci.

[44] Lin N, Chen H, Zhang H, Wan X, Su Q. Mitochondrial reactive oxygen species (ROS) inhibition ameliorates palmitate-induced INS-1 beta cell death. Endocrine. 2012;42:107–17. 10.1007/s12020-012-9633-z 22350662

[45] Smith PK, Krohn RI, Hermanson GT, Mallia AK, Gartner FH, Provenzano MD, Fujimoto EK, Goeke NM, Olson BJ, Klenk DC (1985). Measurement of protein using bicinchoninic acid. Anal Biochem.

[46] Ehrlichová M, Koc M, Truksa J, Naldová Z, Václavíková R, Kovárr J (2005). Cell death induced by taxanes in breast cancer cells: cytochrome C is released in resistant but not in sensitive cells. Anticancer Res.

[47] Voborilova J, Nemcova-Furstova V, Neubauerova J, Ojima I, Zanardi I, Gut I, Kovar J (2011). Cell death induced by novel fluorinated taxanes in drug-sensitive and drug-resistant cancer cells. Invest New Drugs.

[48] Feng XT, Duan HM, Li SL (2017). Protective role of Pollen Typhae total flavone against the palmitic acid-induced impairment of glucose-stimulated insulin secretion involving GPR40 signaling in INS-1 cells. Int J Mol Med.

[49] Ogilvie RI, Nadeau JH, Sitar DS (1982). Diazoxide concentration-response relation in hypertension. Hypertension.

[50] Schütz E, Ha HR, Bühler FR, Follath F (1982). Serum concentration and antihypertensive effect of slow-release verapamil. J Cardiovasc Pharmacol.

[51] Bacracheva N, Thuermann P, Rietbrock N (1990). Dose adjustment of nifedipine in hypertensive patients. Eur J Clin Pharmacol.

