# Supplementary material for: Calcium channel blockers do not protect against saturated fatty acid-induced ER stress and apoptosis in human pancreatic β-cells
Source: Nutr Metab (Lond). 2021 Jul 17;18:74. doi: 10.1186/s12986-021-00597-6 (PMC8285784; doi:10.1186/s12986-021-00597-6)
Supplement: Supplementary file 1 — Additional file 1: Figure S1. In vitro images of NES2Y and 1.1B4 β-cells after the treatment. [file 12986_2021_597_MOESM1_ESM.docx]

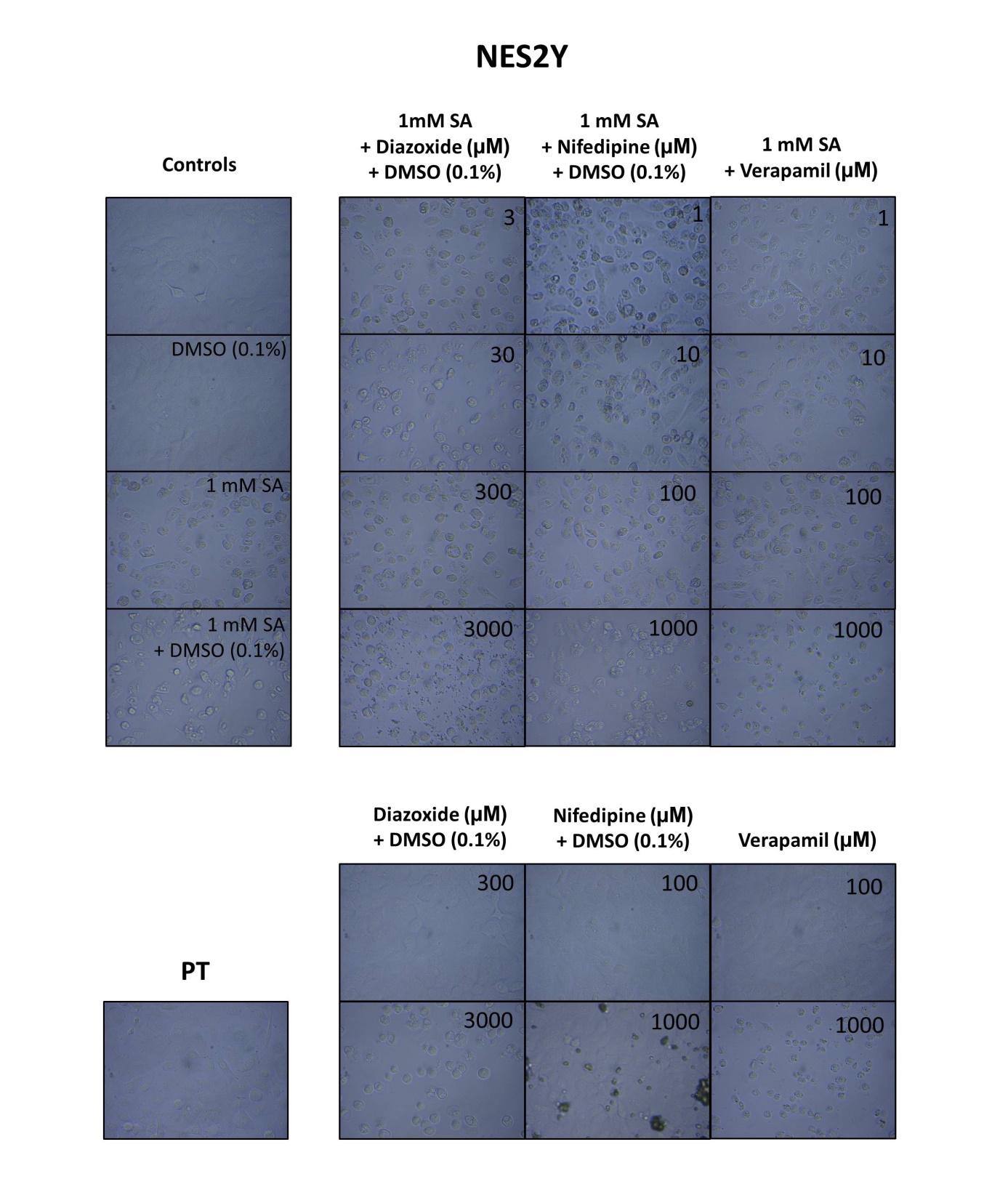
**SUPPLEMENTARY Materials**


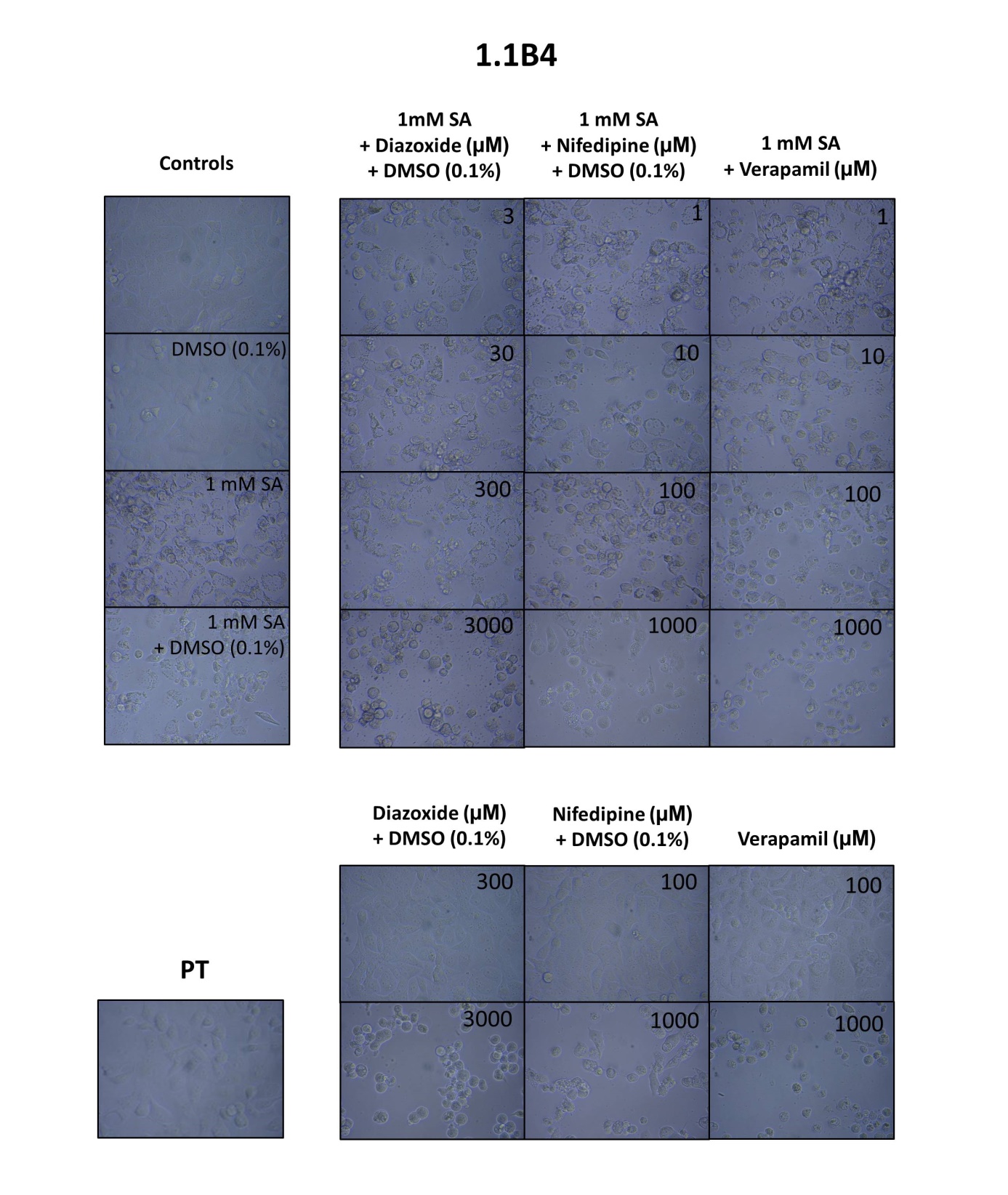


**Figure S1**. *In vitro* images of NES2Y and 1.1B4 β-cells 48 h after the treatment with 1 mM stearic acid (SA) with increasing concentrations of the respective inhibitor, i.e. diazoxide, nifedipine, and verapamil. The image of cells prior to the treatment (PT) is also shown. 20× objective was used.
